# Immunomodulatory Functions of the Human Cathelicidin LL-37 (aa 13–31)-Derived Peptides are Associated with Predicted α-Helical Propensity and Hydrophobic Index

**DOI:** 10.3390/biom9090501

**Published:** 2019-09-18

**Authors:** Mahadevappa Hemshekhar, Sana Faiyaz, Ka-Yee Grace Choi, Oleg V. Krokhin, Neeloffer Mookherjee

**Affiliations:** 1 Manitoba Centre for Proteomics and Systems Biology, Department of Internal Medicine, University of Manitoba, Winnipeg, MB R3E3P4, Canada; Hem.Mahadevappa@umanitoba.ca (M.H.); sanafaiyaz@gmail.com (S.F.); grace@hancocklab.com (K.-Y.G.C.); Oleg.Krokhine@umanitoba.ca (O.V.K.); 2 Department of Immunology, University of Manitoba, Winnipeg, MB R3E 3P4, Canada; 3 Department of Chemistry, University of Manitoba, Winnipeg, MB R3T 2N2, Canada

**Keywords:** cathelicidin, LL-37, IG-19, α-helicity, hydrophobicity, inflammation, immunomodulation, peptide retention time

## Abstract

The anti-endotoxin activity of the cationic peptide LL-37 and its derivative IG-19 is attributed to electrostatic interaction of the peptides’ positive charge with negatively charged bacterial lipopolysaccharides (LPS), and in part to the alteration of intracellular mechanisms independent of peptide binding to LPS. We examined the immunomodulatory responses induced by IG-19 and four IG-19-derived scrambled peptides (IG-19a–d), in the presence and absence of LPS, in macrophages and peripheral blood-derived mononuclear cells. All peptides had identical net charge (+5) and amino acid composition, but different hydrophobicity and α-helical propensity. Peptide IG-19 suppressed LPS-induced cytokine/chemokine production by >90%, IG-19a and IG-19b suppressed it by 40–50%, and IG-19c and IG-19d did not suppress cytokine/chemokine production at all. In silico prediction algorithms and the peptide retention time (RT) on a C18 RP HPLC column indicated a linear association between α-helical propensity and hydrophobicity with the ability of the peptides to inhibit LPS-induced responses. Peptide RT exhibited a significant correlation (>70%) between the suppression of LPS-induced cytokine/chemokine production and peptide-induced production of the anti-inflammatory cytokine IL-1RA. These results indicate that RT on a C18 column can be used as a predictor for the immunomodulatory functions of cationic peptides. Overall, we demonstrated that the immunomodulatory functions of LL-37-derived peptides with identical positive charge and amino acid composition are directly associated with the predicted α-helical propensity and hydrophobicity of the peptides.

## 1. Introduction

Cationic host defense peptides (CHDPs) play a critical role in antimicrobial and immunity-related functions. Cathelicidins and defensins are the most well studied CHDPs in mammals. In humans, the only cathelicidin CHDP is the 37 amino acid peptide LL-37. This peptide is primarily expressed in the granules of neutrophils, in other cell types such as monocytes/macrophages, lymphocytes, epithelial cells, and keratinocytes, and is found in a wide variety of tissues and body fluids [1,2,3]. LL-37 is an immunomodulatory peptide known to mediate both pro- and anti-inflammatory responses [4,5]. LL-37 can facilitate immune-activating functions such as chemokine production, leukocyte recruitment, macrophage and dendritic cell differentiation, and T-cell polarization [5,6,7]. In contrast, LL-37 also mediates anti-inflammatory responses such as suppressing endotoxin-induced pro-inflammatory cytokines and intervening in toll-like receptor (TLR)-induced signaling in the presence of pathogenic ligands [8]. We have previously shown that LL-37 can also suppress inflammatory responses in the absence of microbial pathogens, such as the response induced by the cytokine IL-32, while inducing the production anti-inflammatory cytokines e.g., interleukin-1-receptor antagonist (IL-1RA) and IL-10 [9,10,11,12]. The mechanisms that regulate the dichotomy of pro- and anti-inflammatory responses mediated by LL-37 and its derivative peptides are not completely understood [11]. Furthermore, the relationship between LL-37-mediated immunomodulatory functions and its sequence and/or biochemical properties has not been completely defined.

The minimum region of LL-37 required for its immunomodulatory functions is an internal sequence from amino acids 13–31 [13], known as peptide IG-19. Similar to LL-37, IG-19 exhibits both antimicrobial and immunomodulatory functions [10,13,14]. IG-19 suppresses inflammatory cytokine production by engaging various signaling intermediates in the presence of bacterial endotoxin or cytokine IL-32 [10,13,14]. Both LL-37 and IG-19 also share structural similarities; these are both α-helical amphipathic peptides with net positive charges of +6 and +5, respectively [13,14]. Previous studies have suggested that the ability of cationic peptides such as LL-37 to suppress bacterial lipopolysaccharide (LPS)-induced pro-inflammatory responses is largely due to peptide binding to negatively charged LPS through electrostatic interactions [15,16]. However, suppression of LPS-induced pro-inflammatory responses by LL-37 is not solely dependent on peptide binding to LPS, as it also involves the ability of the peptide to intervene in TLR-to-NF-κB signaling mechanisms [8,12,17]. Moreover, both LL-37 and IG-19 can suppress inflammatory responses in the absence of LPS, and can activate specific anti-inflammatory signaling pathways such as the one mediated by the regulatory dual phosphatase MKP-1 [10]. Therefore, it can be speculated that the immunomodulatory functions of LL-37 and its derivative cationic peptides may be in part independent of its net positive charge and dependent on other biochemical properties.

In this study, we examined the relationship between certain structural characteristics and the immunomodulatory functions of IG-19 and four of its scrambled peptides in human mononuclear cells. All peptides had the same amino acid composition and net positive charge of +5 (Table 1). We demonstrated that although all the peptides had an identical net positive charge, their ability to suppress LPS-induced cytokine and chemokine production, as well as peptide-mediated cytokine/chemokine production, varied significantly. We also demonstrate that these immunomodulatory responses are directly associated with the predicted α-helicity and hydrophobicity of the peptide. We further showed that the retention time (RT) of the peptides on a C18 Reversed Phase High Performance Liquid Chromatography (RP HPLC) column varied significantly, and that the peptide-mediated immunomodulatory functions correlated significantly with RT in both a macrophage-like cell line and in primary human peripheral blood mononuclear cells (PBMC). Results of this study suggest that net positive charge is not the sole determinant of the immunomodulatory functions of cationic CHDP. Based on the results of this study, we propose that peptide RT may be a useful tool with which to screen and predict immunomodulatory functions of CHDP and related synthetic derivatives.

## 2. Results

### 2.1. Peptides’ Amphipathicity and Predicted Structural Properties

**Figure 1:**
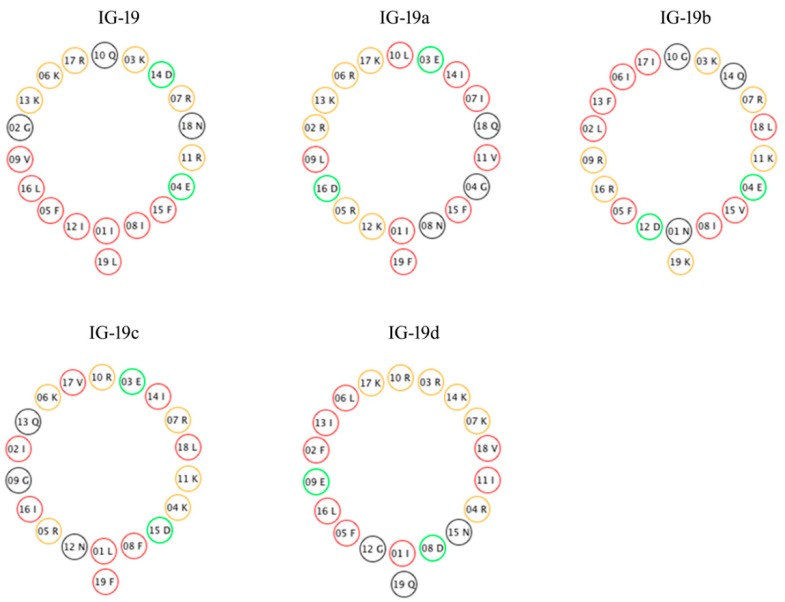
Helical projections of the peptides. The hydrophobic amino acids are indicated as red rings, hydrophilic basic amino acids (positively charged) are indicated in yellow, hydrophilic acidic amino acids (negatively charged) are indicated in green rings, and all other amino acids are represented in black rings.

Peptide IG-19 and the four IG-19-derived scrambled analogs used in this study had identical amino acid composition and net positive charge, +5 at pH 7.4 and +7 at pH 2 (Table 1). The α-helical wheel projections showed that the IG-19 peptide was amphipathic with clearly defined hydrophobic and hydrophilic faces of its helix (Figure 1).

The selection of the four IG-19-derived scrambled peptides for this study was based on in silico secondary structure and hydrophobicity predictions, aiming to provide wide range variation of these parameters (Table 2). Peptide IG-19 showed the highest percentage of α-helicity (>80%), followed by peptides IG-19a (>55%) and IG-19b (>40%), and peptides IG-19c and IG-19d had less than 40% α-helicity (Table 2). Hydrophobicity index (HI, acetonitrile % units) predicted using SSRCalc determined IG-19 as the most hydrophobic (HI, 11.15), followed by IG-19a (HI, 9.4), IG-19b (HI, 10.95), IG-19c (HI, 8.87), and IG-19d with least hydrophobicity (HI, 7.59) (Table 2). Therefore, the peptides used in this study had identical composition and net positive charge, but varied in α-helicity and hydrophobicity, with IG-19 demonstrating the highest and IG-19d the least α-helical propensity and HI (Table 2).

### 2.2. Cytokine and Chemokine Production in Macrophage-like THP-1 Cells

**Figure 2:**
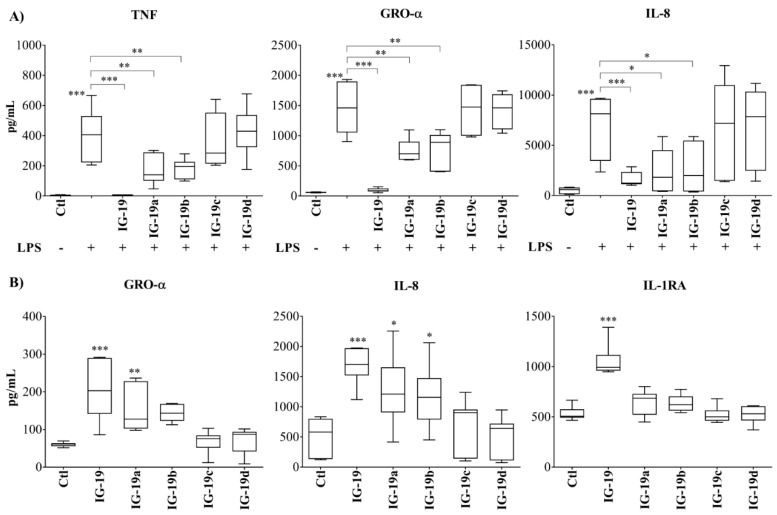
Cytokine and chemokine production in macrophage-like THP-1 cells. Macrophage-like plastic adherent THP-1 cells were stimulated with lipopolysaccharides (LPS) (10 ng/mL) in the presence and absence of different peptides (5 µM each). Production of pro-inflammatory cytokine TNF and chemokines GRO-α and IL-8 were evaluated by ELISA in tissue culture (TC) supernatants after 24 h, and that of anti-inflammatory cytokine IL-1RA after 48 h. (**A**) LPS-induced responses (TNF, GRO-α, and IL-8) in the presence and absence of the peptides. (**B**) Peptide-induced production of chemokines (GRO-α and IL-8) and anti-inflammatory cytokine IL-1RA. Results shown are from eight independent experimental replicates. Results shown are from seven independent experiments (*n* = 7). One-way analysis of variance (ANOVA) followed by Tukey’s *post hoc* test was used to determine the statistical significance. A *p*-value of <0.05 was considered to be statistically significant.

Previous studies have shown that IG-19 (5 µM) significantly suppresses LPS-induced pro-inflammatory cytokine and chemokine production, and induces the production of the anti-inflammatory cytokine IL-1RA in macrophage-like THP-1 cells [10,13,14]. Therefore, we compared the effects of IG-19 and the four scrambled IG-19-derivative peptides on cytokine and chemokine production in the presence and absence of LPS. Macrophage-like THP-1 cells were pre-incubated with the peptides (5 µM each) for 30 min prior to stimulation with bacterial LPS (10 ng/mL). Production of pro-inflammatory cytokine TNF and chemokines (GRO-α and IL-8) were measured in tissue culture (TC) supernatants after 24 h, and that of IL-1RA after 48 h. Consistent with previous studies, IG-19 suppressed LPS-induced pro-inflammatory cytokine and chemokine production by >90% (Figure 2A), whereas peptides IG-19a and IG-19b inhibited LPS-induced TNF and chemokines 40–50%, and in contrast, peptides IG-19c and IG-19d did not inhibit LPS-induced responses (Figure 2A). Aligned with this, peptide IG-19 significantly induced IL-1RA, followed by modest induction by IG-19a and IG-19b, whereas peptides IG-19c and IG-19d did not induce the production of IL-1RA (Figure 2B). A similar response was also observed with peptide-induced chemokine production, where IG-19 significantly induced Gro-α and IL-8, followed by IG-19a and IG-19b, and the peptides IG-19c and IG-19d did not significantly induce chemokines in macrophage-like THP-1 cells (Figure 2B).

**Figure 3:**
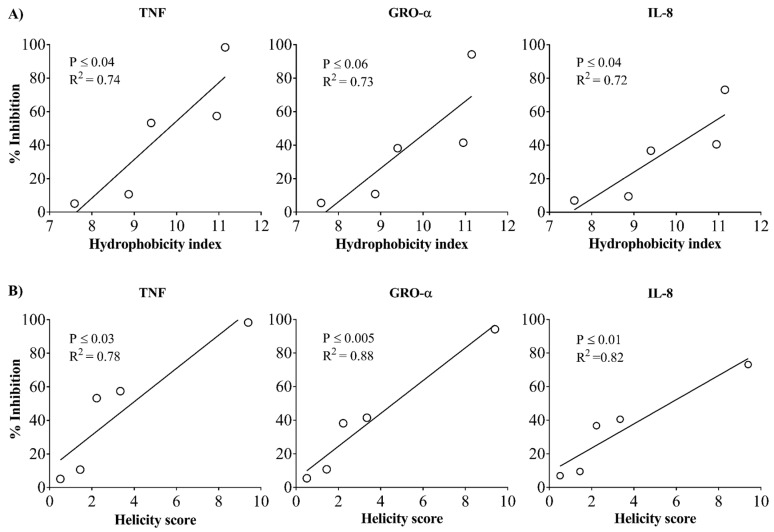
Correlation analyses of immunomodulatory responses of peptides with in-silico-predicted hydrophobicity index and α-helicity scores. Macrophage-like plastic adherent THP-1 cells were stimulated with LPS (10 ng/mL) in the presence and absence of the different peptides (5 µM each). Production of pro-inflammatory cytokine TNF and chemokines GRO-α and IL-8 was evaluated by ELISA in TC supernatants after 24 h (**A**) Correlation analysis between the percentage inhibition of LPS-induced responses and predicted hydrophobicity index (HI) of the peptides. (**B**) Correlation analysis between the percentage inhibition of LPS-induced responses and predicted α-helicity scores (AGADIR prediction) of the peptides. Each ring in the graph represents an individual peptide. Pearson correlation analysis was used to determine the significance of the correlation. A *p*-value of <0.05 was considered to be statistically significant.

These results show that although all the peptides had an identical net charge, their ability to suppress LPS-induced cytokine and chemokine secretion, and induce anti-inflammatory cytokine IL-1RA and chemokines varied significantly. Correlation analyses demonstrated a linear association between peptide-mediated suppression of LPS-induced cytokine production (TNF, GRO-α, and IL-8) with predicted HI (Figure 3A) and α-helicity scores of the peptides (Figure 3B).

### 2.3. Peptide Retention Time (RT) on C18 Reversed-Phase (RP) HPLC Column

**Figure 4:**
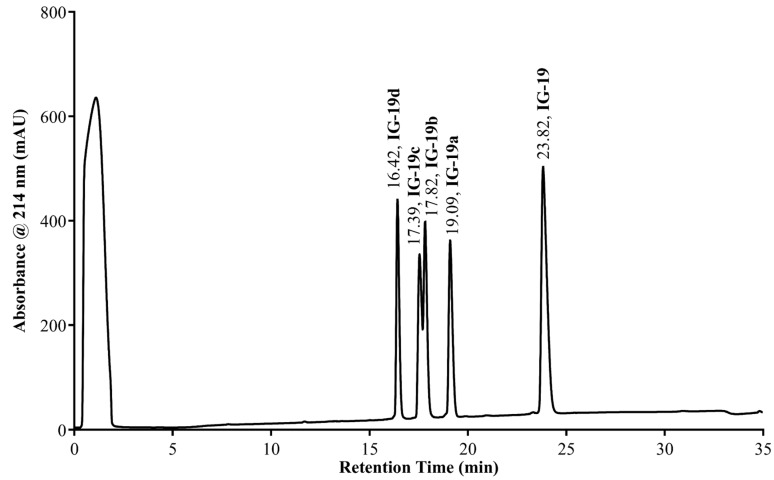
RP HPLC separation of IG-19 and its scrambled analogs. The mixture of IG-19 and its scrambled analogs (5 µg of each) was injected on the Luna C18(2) 5 µm, 1 × 100 mm column Phenomenex (Torrance, CA) and separated using a binary water/acetonitrile gradient of 2% acetonitrile per minute (0.1% trifluoroacetic acid as ion-pairing modifier) with 0.2 mL/min flow rate.

RP HPLC is a powerful tool for measuring the hydrophobicity of individual amino acids and establishing the presence of preferentially interacting domains between the peptide and hydrophobic surfaces [18,19]. It has been previously used to delineate the antimicrobial mechanism of action of amphipathic peptides [20]. It was previously noted that the C18-bonded chemistry of RP HPLC sorbents mimics biomembranes and can be used as an artificial analog to study peptide interactions with hydrophobic surfaces [21,22]. It is widely acknowledged that the amphipathic character of peptides leads to increased RT related to hydrophobicity in RP HPLC systems. We found that the RT of the peptides on the C18 column varied significantly. Peptide IG-19 had the highest RT of 23.82 min, followed by IG-19a (19.09 min), IG-19b (17.82 min), IG-19c (17.39 min), and IG-19d (16.42 min) (Figure 4).

### 2.4. Association of Peptides’ Immunomodulatory Functions with Retention Properties

**Figure 5:**
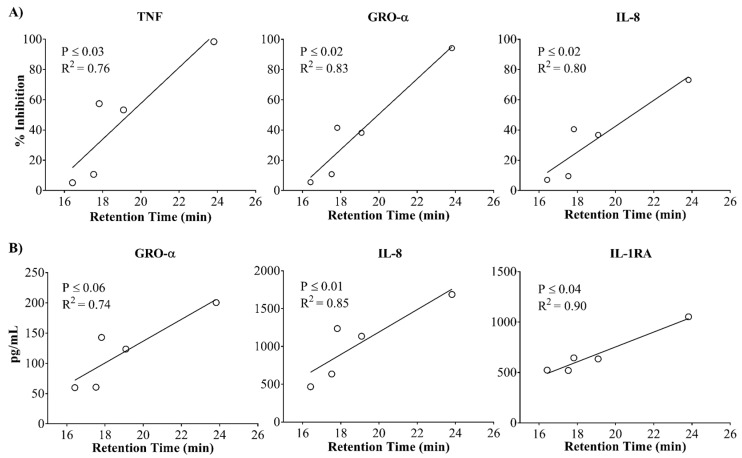
Correlation analyses of immunomodulatory responses of peptides with retention time (RT). Macrophage-like plastic adherent THP-1 cells were stimulated with LPS (10 ng/mL) in the presence and absence of the different peptides (5 µM each). Production of pro-inflammatory cytokine TNF and chemokines GRO-α and IL-8 were evaluated by ELISA in TC supernatants after 24 h, and that of anti-inflammatory cytokine IL-1RA after 48 h. (**A**) Correlation analysis between the percentage inhibition of LPS-induced responses by peptides and RT of the peptides on RP HPLC. (**B**) Correlation analysis between the peptide-induced chemokines (GRO-α and IL-8) and anti-inflammatory cytokine IL-1RA, and RT of the peptides on RP HPLC. Each ring in the graph represents an individual peptide. Pearson correlation analysis was used to determine the significance of the correlation. A *p*-value of <0.05 was considered to be statistically significant.

We demonstrated a correlation between the observed RT of the peptides on the C18 column and their biological activity (Figure 5). Percent inhibition of LPS-induced TNF, GRO-α, and IL-8 in presence of the peptides showed significant linear correlation with the RT, with R^2^ values of 0.76, 0.83, and 0.80, respectively (Figure 5A). Similarly, peptide-induced GRO-α, IL-8, and IL-1RA production also exhibited statistically significant linear correlation with peptide RT, with R^2^ values of 0.74, 0.85, and 0.90, respectively (Figure 5B).

### 2.5. Peptide-Mediated Responses in Human Peripheral Blood Mononuclear Cells (PBMC)

**Figure 6:**
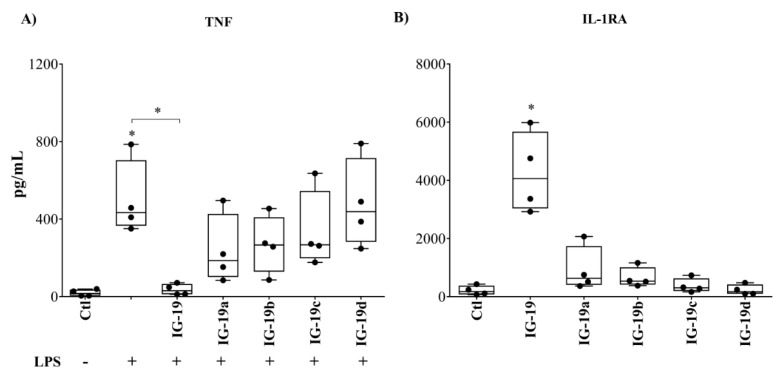
Functional validation of the peptide immunomodulatory responses using human peripheral blood mononuclear cells (PBMC). Human PBMC were stimulated with LPS (10 ng/mL) in the presence and absence of different peptides (5 µM each). (**A**) LPS-induced production of pro-inflammatory cytokine TNF in the presence or absence of peptides was evaluated by ELISA in TC supernatants after 24 h. (**B**) Peptide-induced production of anti-inflammatory cytokine IL-1RA was evaluated by ELISA in TC supernatants after 48 h. Results shown are from four biological replicates using PBMC isolated from four independent donors (*n* = 4). Each dot represents an independent donor and the line shown is the median for each condition. Kruskal–Wallis one-way analysis of variance (ANOVA) followed by Dunn’s *post hoc* test was used to determine the statistical significance. * A *p*-value of <0.05 was considered to be statistically significant.

To confirm the results related to the anti-inflammatory activity of the peptides observed in macrophage-like THP-1 cells in primary cells, we further examined the production of cytokines TNF and IL-1RA in human PBMC. The cells were stimulated with the different peptides (5 µM each) in the presence or absence of bacterial LPS (10 ng/mL). Production of TNF was examined in TC supernatants by ELISA after 24 h, and that of IL-1RA after 48 h. Peptide IG-19 mitigated LPS-induced TNF production by >90%, whereas peptides IG-19a and IG-19b partially inhibited LPS-induced TNF production (~30%), and peptides IG-19c and IG-19d did not significantly suppress LPS-induced TNF production in PBMC (Figure 6A). Peptide IG-19 significantly induced IL-1RA production, whereas none of the other peptides induced IL-1RA in PBMC (Figure 6B).

**Figure 7:**
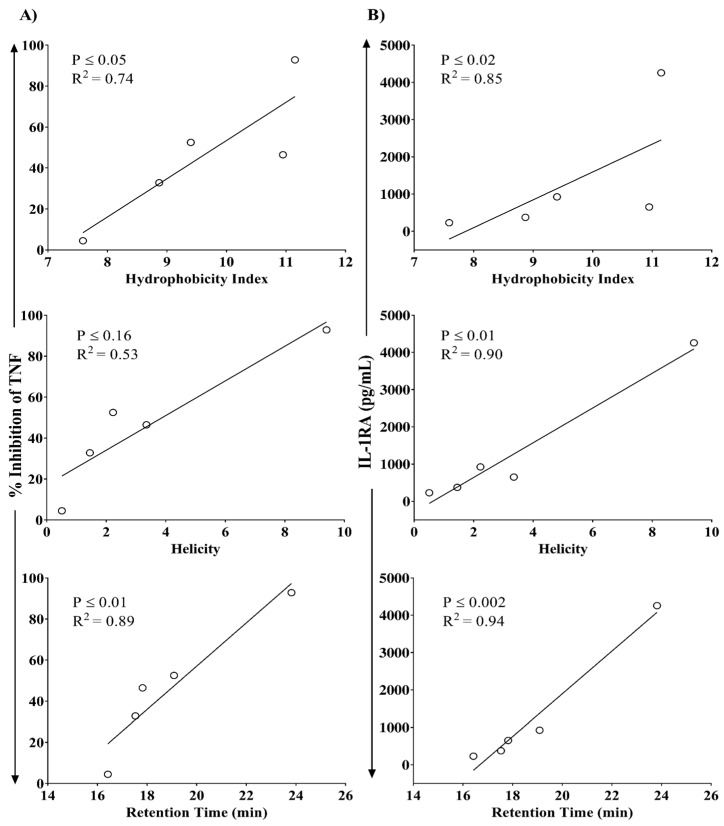
Correlation analyses of peptide-mediated activity in human PBMC with in-silico-predicted hydrophobicity index (HI), α-helicity scores, and retention time (RT). Human PBMC were stimulated with LPS (10 ng/mL) in the presence and absence of the different peptides (5 µM each). Production of pro-inflammatory cytokine TNF was evaluated by ELISA in TC supernatants after 24 h, and IL-1RA after 48 h. Correlation analysis between predicted HI, helicity, and RT with peptide-mediated (**A**) percentage inhibition of LPS-induced TNF and (**B**) IL-1RA production. Each ring in the graph represents an individual peptide. Pearson correlation analysis was used to determine the significance of the correlation. A *p*-value of <0.05 was considered to be statistically significant.

Correlation analyses showed a significant linear association between the ability of the peptides to suppress LPS-induced TNF production in PBMC with predicted HI and RT of the peptides on C18 column, with R^2^ values of 0.74 and 0.89, respectively (Figure 7A). Peptide-mediated production of IL-1RA in PBMC significantly correlated with predicted HI, helicity, and RT of the peptide, with R^2^ values of 0.85, 0.90, and 0.94, respectively (Figure 7B).

## 3. Discussion

Limited studies have explored the structure-to-function relationship of the human CHDP LL-37 in the context of immunomodulation. Peptide IG-19, representing an internal sequence of LL-37 (amino acids 13–31), is the minimum region required for the immunomodulatory functions of LL-37 [4,13]. Similar to LL-37, IG-19 suppresses LPS-induced inflammatory responses in human monocytic cells and in corneal fibroblasts [13,14,23,24]. Previous studies have suggested that maintaining the integrity of the secondary structure of LL-37 and IG-19 is required to induce cellular responses [13,25]. The ability of these peptides to suppress LPS-induced responses is thought to be primarily mediated by the electrostatic interaction of the positive charge on the peptide with negatively charged LPS [26,27], with an increase in cationic charge being related to stronger binding and neutralization of LPS-induced cellular responses [15,28,29]. However, in this study we demonstrated that IG-19 and its derivative peptides, all with identical net positive charge (+5 at pH 7.4), varied significantly in their ability to suppress LPS-induced cellular responses. We further showed that suppression of LPS-induced cytokine/chemokine secretion by the cationic peptides was directly associated with predicted amphipathic helicity and hydrophobic interactions with RP chromatographic sorbent, assessed by the RT of the peptides. The results of this study suggest that electrostatic binding of the cationic peptides to LPS may not be the primary mode of action in suppressing endotoxin-induced cellular responses. This is consistent with previous studies showing that regulation of LPS-induced cellular responses by LL-37 and IG-19 may not be entirely dependent on direct LPS binding by the peptides [8,13,23]. The anti-inflammatory activities of LL-37 and IG-19 have been shown to be mediated by mechanisms that involve the regulation of the AKT–MAPK signaling pathway and activation of the dual phosphatase MKP-1, which is a negative regulator of inflammation, and by the promotion of anti-inflammatory cytokines such as IL-1RA in myeloid cells, in the absence of LPS [10]. Consistent with this, here we have demonstrated that the ability of IG19-derived peptides with identical charges to induce IL-1RA varied significantly compared to IG-19 in both a macrophage-like cell line and primary human PBMC. A recent study demonstrated that a cathelicidin-derived synthetic cationic peptide suppresses inflammatory responses in a murine model of sterile inflammation [30], demonstrating the anti-inflammatory activity of the peptide in the absence of endotoxin in vivo. Taken together, these studies suggest that while the anti-inflammatory functions of cationic peptides, including their anti-endotoxin activity, involve electrostatic interaction of the peptides with stimulants such as LPS, these interactions do not play a role in determining the strength of the immunomodulatory response. This may be due to the involvement of other cellular components that contribute to peptide-mediated immunomodulatory mechanisms of action in vivo.

In this study, we have shown that there is a significant correlation between the immunomodulatory responses mediated by CHDP-derived peptides with the in silico predicted α-helicity and hydrophobicity of the peptides. This is consistent with previous studies demonstrating that the helicity of peptides plays a key role in peptide specificity and biological activity [31]. Increasing the helical propensity of peptides by replacing either hydrophobic or hydrophilic amino acids has been shown to enhance specificity of the antimicrobial functions of cationic peptides, thus suggesting the use of helicity as a rationale for designing and optimizing antimicrobial peptides [31,32]. Similarly, based on the results of this study, it can be speculated that structural characteristics, namely α-helicity and hydrophobicity, could be used to predict immunomodulatory functions for cationic peptides such as those derived from cathelicidin CHDP.

Furthermore, we demonstrated that the immunomodulatory activity of IG-19 and its derivative peptides exhibits a strong correlation with peptide retention in RP HPLC. The retention behavior of peptides in RP HPLC is a powerful tool with which to elucidate the apparent hydrophobicity of the peptides, which depends on both amino acid composition and the conformational status of peptides interacting with hydrophobic C18 surface [33,34]. Previous studies have also shown the effects of amino acid substitution in peptide α-helical regions on their retention properties [21]. The nonpolar region of an amphipathic α-helical peptide is the preferred binding domain in interactions with the hydrophobic matrix of a RP column, and this interaction defines peptide RP HPLC retention [20,22,35]. Therefore, RT on a C18 column is associated with both hydrophobicity and α-helicity of peptides. RP HPLC has been previously used to characterize the apparent hydrophobicity and helicity of cationic antimicrobial peptides [33]. The results of this study suggest that the immunomodulatory activity of CHDP-derived peptides is associated with hydrophobicity and helicity, which correlates with RT on a C18 RP HPLC column. Consistently with this, hydrophobicity of CHDP has been shown to strongly correlate with peptide-mediated mitigation of TLR-dependent pro-inflammatory responses, including those activated by LPS [13]. However, it should be noted that using C18 media to understand the behavior of peptides upon interaction with biomembranes [21,22] represents an approximation only. Phosphatidylcholine bilayers representing cell membranes possess additional outer zwitterionic functionality, providing extra complexity. Therefore, chromatographic columns carrying phosphatidylcholine functional groups would be useful in accurately delineating the interactions of amphipathic cationic peptides with biological membranes. For example, the IAM PC (immobilized artificial membrane phosphatidyl choline) column has been suggested for the study of cell permeability for drug candidates [36]. The first study on peptide behavior using the IAM PC column confirmed similarity in the retention of amphipathic helical peptides with C18 phases [37]. However, it was also observed that positively charged amino acids (Lys, Arg, His) provided a higher contribution to retention on an IAM PC compared to C18, due to stronger interaction of lipid-absorbed peptides with the inner components of the zwitterionic layer—negatively charged phosphate groups. This complexity may explain some of the discrepancies in this study, that is, even though the immunomodulatory activity of some of the peptides correlated significantly with their hydrophobic index, helicity score, and RT, their predictive scores (hydrophobicity and helicity) did not exactly correlate with the RT on the C18 column. Therefore, the IAM PC column could be used in future studies to further investigate the relationship between the chromatographic properties and immunomodulatory functions of cationic peptides derived from CHDP. Nevertheless, based on the results of this study, we propose that the RT of peptides could be used as a screening tool to assess the immunomodulatory activity of peptides derived from CHDP.

In conclusion, in this study we demonstrated that the immunomodulatory functions of CHDP-derived synthetic peptides, such as inhibition of LPS-induced inflammatory cytokines and induction of the anti-inflammatory cytokine IL-1RA and chemokines, exhibit strong correlations with the predicted α-helicity and hydrophobicity of the peptides. We demonstrated that the experimental RT of the peptides on a C18 RP HPLC column also correlates with the immunomodulatory functions of the peptides, further highlighting the effectiveness of the existing in silico predictive algorithms used in this study. Overall, the results suggest that the immunomodulatory functions of CHDP and its derivative peptides may depend largely on structural features such as amphipathic helicity and are not solely dependent on the net positive charge. Based on the results of this study, we propose that predicted and experimentally measured peptide retention values on a C18 RP HPLC column could be a potential tool for screening CHDP-derived synthetic peptides and predicting their immunomodulatory functions.

## 4. Methods

### 4.1. Peptides and Other Reagents

All peptides (Table 1) were synthesized by CPC Scientific (Sunnyvale, CA, USA), dissolved in endotoxin-free water, aliquoted, and stored at −20 °C until use. *Escherichia coli* LPS was obtained from Sigma-Aldrich (Oakville, ON, Canada). Luna C18(2) 5 µm, Phenomenex, 1 × 100 mm column was obtained from Phenomenex (Torrance, CA, USA). Specific antibody pairs for ELISA for the detection of TNF were obtained from eBioscience (San Diego, CA, USA), and for GRO-α, IL-8, and IL-1RA, were obtained from R&D systems (Minneapolis, MN, Canada).

### 4.2. Peptide Design and In Silico Determination of Helicity and Hydrophobicity Index

Using the sequence of peptide IG-19 (amino acids 13–31 of LL-37), four other peptides (IG-19a, IG-19b, IG-19c, and IG-19d) were generated by scrambling the position of the amino acids. In silico predictions of the α-helicity of these peptides (Table 1) were made using a prediction algorithm, Agadir-A software (http://agadir.crg.es/about.jsp), based on the helix/coil transition theory to predict the helical content of the peptides in this study. We used the Agadir online tool to calculate the helical content of peptides in water solutions at pH 7, ionic strength 0.1. The PredictProtein—Protein Sequence Analysis software was used to predict the structural features of the peptides (https://www.predictprotein.org/). This is open source software that has been used for prediction of protein/peptide structural components in previous studies [38,39]. The hydrophobicity index (HI, acetonitrile percentage units) for the peptides was determined using the Sequence Specific Retention Calculator (SSRCalc), (http://hs2.proteome.ca/SSRCalc/SSRCalcQ.html; Version Q point oh ©2015 at the Manitoba Centre for Proteomics & Systems Biology (Winnipeg, MB, Canada)) [40].

### 4.3. Cell Culture

Human monocytic THP-1 cells obtained from American Type Culture Collection (ATCC^®^ TIB-202) were cultured in RPMI 1640 medium containing 1 mM sodium pyruvate and 10% (*v*/*v*) fetal bovine serum (FBS), referred to as complete RPMI medium hereafter. The cells were maintained at 37 °C in a 5% CO_2_ humidified incubator. Cells were seeded at a density of 1.1 × 10^5^ cells/cm^2^ in 24 well tissue culture (TC) plates and treated with 50 ng/mL PMA for 24 h to obtain plastic-adherent macrophage-like cells, as previously described by us [10,11]. Macrophage-like plastic-adherent THP-1 cells were rested in fresh complete RPMI 1640 medium for an additional 24 h before stimulation. These cells were stimulated with the different peptides (5 μM each) as indicated, in the presence and absence of LPS (10 ng/mL). The concentrations of the different stimulants were based on our previous studies [10,11,17]. TC supernatants were centrifuged at 250× *g* for 5 min to obtain cell-free supernatants, aliquoted, and stored at −20 °C until use [10,11].

### 4.4. Human Peripheral Blood Mononuclear Cell (PBMC) Isolation

Venous blood was collected from healthy volunteers with written informed consent, according to a protocol approved by the Health Research Ethics Board at the University of Manitoba (protocol number H2010:259 (HS11105). Blood was diluted with complete RPMI medium (1:1) and fractionated over Ficoll–Paque Plus (GE Healthcare Life Sciences, Baie d’Urfe, Quebec, Canada). The buffy coat was isolated and washed twice in complete RPMI medium (300× *g* for 10 min). PBMC were isolated from the buffy coat as previously described by us [8,10,11]. PBMC were seeded (1 × 10^6^ cells/mL/well) into 24 well TC plates and rested for 2 h at 37 °C in a humidified 5% CO_2_ incubator before stimulation. TC supernatants were collected, centrifuged at 250× *g* for 5 min to obtain cell-free supernatants, aliquoted, and stored at −20 °C until use.

### 4.5. Enzyme-Linked Immunosorbent Assay (ELISA)

Production of the cytokine TNF was measured in TC supernatants by ELISA using specific antibody pairs obtained from eBioscience (San Diego, CA, USA), as per the manufacturer’s instructions. Production of cytokine IL-1RA and chemokines GRO-α and IL-8 was evaluated in the TC supernatants using antibody pairs from R&D Systems as per the manufacturers’ instructions. Serial dilutions of the recombinant human cytokines or chemokines were used to establish a standard curve for evaluation of the concentrations in the TC supernatants.

### 4.6. RP HPLC

An Agilent 1100 series HPLC system with UV detection at 214 nm and manual 50 uL injector was used for chromatographic experiments. Samples containing 5 µg of individual peptides or their mixtures were injected into the Luna C18(2) 5 µm, 1 × 100 mm column Phenomenex (Torrance, CA, USA) and separated using binary water/acetonitrile gradient of 2% acetonitrile per minute (0.1% trifluoroacetic acid as ion-pairing modifier) with a 0.2 mL/min flow rate.

### 4.7. Statistical Analyses

GraphPad Prism 7.02 software (GraphPad software, San Diego, CA, USA; The product was licensed through University of Manitoba for research use) was used for statistical analyses. One-way analysis of variance (ANOVA) followed by Tukey’s *post hoc* test was used to determine the statistical significance. Correlation analyses between RT on the C18 RP HPLC column and peptide activity were performed by Pearson’s correlation analysis. A *p*-value of <0.05 was considered to be statistically significant.

## Figures and Tables

**Table 1:** Amino acid sequences and net charge of the peptides.

Peptides	Sequence	Net Charge at pH 7	Net Charge at pH 2
IG19	IGKEFKRIVQRIKDFLRNL-NH2	+5	+7
IG-19a	IREGRRINLLVKKIFDKQF-NH2	+5	+7
IG-19b	NLKEFIRIRGKDFQVRILK-NH2	+5	+7
IG-19c	LIEKRKRFGRKNQIDIVLF-NH2	+5	+7
IG-19d	IFRRFLKDERIGIKNLKVQ-NH2	+5	+7

**Table 2:** In silico prediction of peptide structural features.

Peptides	Secondary Structure Score (PredictProtein Software)			α- Helicity Score (AGADIR)	Hydrophobicity Index (Predicted)
	Helix	Strand	Loop		
IG19	84.2%	0.0%	15.8%	9.4	11.15
IG-19a	57.9%	0.0%	42.1%	2.23	9.40
IG-19b	42.1%	21.1%	36.8%	3.35	10.95
IG-19c	35.6%	15.8%	48.6%	1.45	8.87
IG-19d	39.1%	0.0%	59.9%	0.51	7.59
